# Supplementary material for: Fullerenes on a Nanodiamond Platform Demonstrate Antibacterial Activity with Low Cytotoxicity
Source: Pharmaceutics. 2023 Jul 19;15(7):1984. doi: 10.3390/pharmaceutics15071984 (PMC10383838; doi:10.3390/pharmaceutics15071984)
Supplement: Supplementary file 1 [file pharmaceutics-15-01984-s001.zip › pharmaceutics-2491984-supplementary.pdf]

Supplementary materials

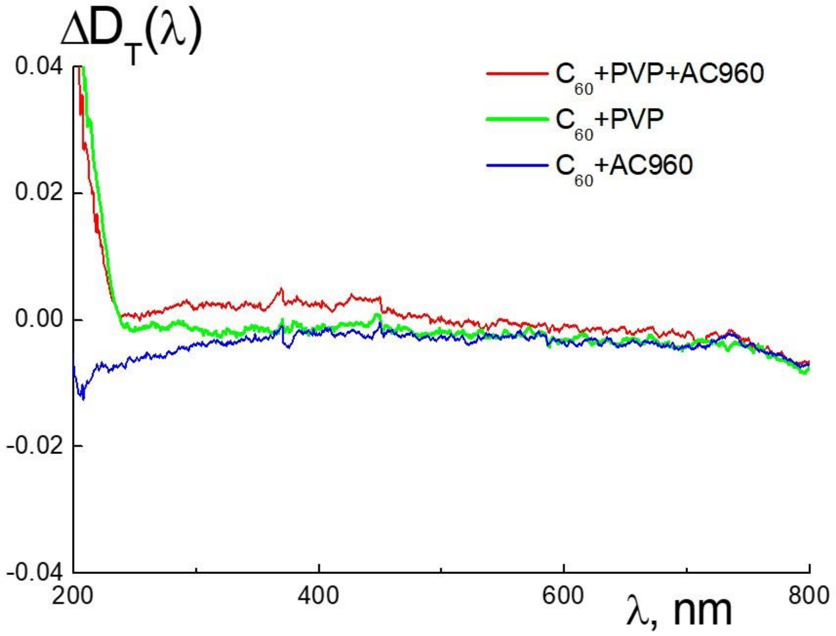

Figure S1. Differences $\Delta D_T(\lambda) = D(\lambda, T{=}50^oC) - D(\lambda, T_I)$ between optical density values vs. light wavelength at 50ºC and initial temperature for aqueous solutions of ternary and binary complexes.

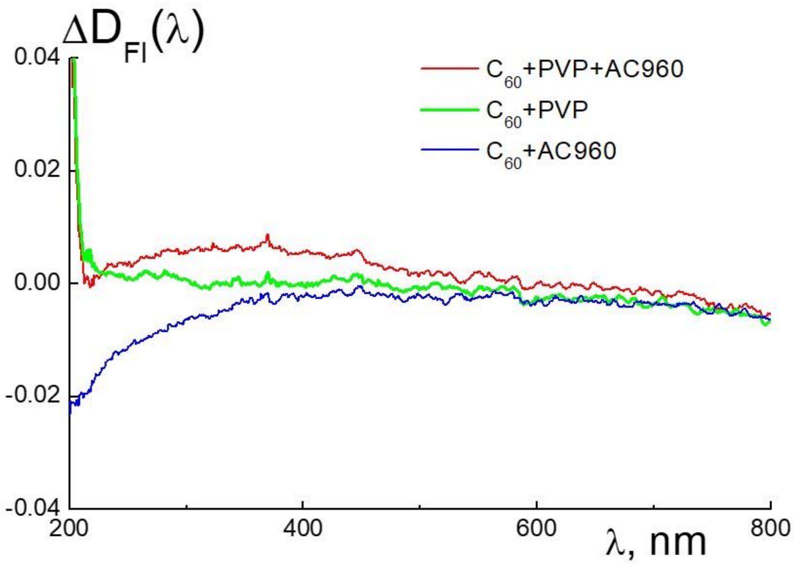

Figure S2. Differences between optical density values $\Delta D_{FI}(\lambda) = D(\lambda, T_F) - D(\lambda, T_I)$ vs. light wavelength for aqueous solutions of ternary and binary complexes at final (T_F = 25ºC) and initial temperature (T_I = 23-24ºC).
